# Phylogeny and Taxonomy of Spider-Pathogenic *Gibellula* (Cordycipitaceae, Hypocreales) from the Lancang–Mekong Biodiversity Hotspot: Four New Species and Five New National Records

**DOI:** 10.3390/jof12050357

**Published:** 2026-05-12

**Authors:** Bo Tu, Hui Chen, Xu Zhang, De-Xiang Tang, Van-Minh Dao, Chanhom Loinheuang, Yao Wang

**Affiliations:** 1 State Key Laboratory of Functions and Applications of Medicinal Plants, Guizhou Medical University, Guian New District, Guiyang 561113, China; tb3318@gmc.edu.cn; 2 State Key Laboratory of Discovery and Utilization of Functional Components in Traditional Chinese Medicine, School of Pharmaceutical Sciences, Guizhou Medical University, Guian New District, Guiyang 561113, China; chui68769@gmail.com (H.C.); xuzhanggmu2018@163.com (X.Z.); tangdx1516@163.com (D.-X.T.); 3 The High Efficacy Application of Natural Medicinal Resources Engineering Center of Guizhou Province, Guizhou Medical University, Guian New District, Guiyang 561113, China; 4 Institute of Regional Research and Development, Ministry of Science and Technology, Hanoi 100803, Vietnam; minhdaovan87@gmail.com; 5 Department of Biology, Faculty of Natural Sciences, National University of Laos, Vientiane 01080, Laos; chanhoml24@gmail.com

**Keywords:** new species, spider-pathogenic fungi, *Gibellula*, taxonomy, Lancang–Mekong Basin

## Abstract

*Gibellula* (Cordycipitaceae, Hypocreales) represents a group of highly specialized obligate fungal pathogens restricted to spider hosts. Species delimitation was conducted using morphological characteristics in combination with multilocus phylogenetic analyses (nr*SSU*, ITS, nr*LSU*, *tef1-α*, *rpb1*, *rpb2*), and we recognized nine spider-associated *Gibellula* species from specimens collected in the Lancang–Mekong biodiversity hotspot (China, Laos, Thailand, and Vietnam). Among them, four are described as new to science: *Gibellula longiconidiophora* sp. nov., *G. mekongensis* sp. nov., *G. ovorum* sp. nov., and *G. pseudopilosa* sp. nov. The other five species represent new national distributional records: *G. yunnanensis* (new to Laos), *G. pseudopigmentosa* (new to Thailand), *G. trimorpha* (new to Vietnam), *G. penicillioides* (new to Laos), and *G. scorpioides* (new to China and Laos). Phylogenetic analyses resolved these taxa into well-supported lineages. Notably, *G. ovorum* is a rare example of a *Gibellula* species parasitizing spider egg sacs rather than adult spiders, revealing an unusual substrate shift. Morphological distinctions among the new species include differences in conidiophore length, synnematal development, conidial size, and sporulation patterns. Detailed descriptions, illustrations, and taxonomic comparisons are provided. This study significantly expands the known diversity and geographic distribution of *Gibellula* in the Lancang–Mekong region and underscores the importance of integrative taxonomy for uncovering hidden diversity in spider-pathogenic fungi.

## 1. Introduction

Entomopathogenic fungi are key components of terrestrial ecosystems, acting as natural antagonists that regulate arthropod population dynamics. A defining feature of these fungi is their capacity to manipulate host behavior, an adaptive strategy that optimizes fungal propagation, spore dispersal, and horizontal transmission. Many fungal taxa cause infected hosts to die in microhabitats that are physiologically and spatially favorable for fungal colonization and reproduction [1,2,3]. A well-documented example is the manipulation of arthropods to die in an elevated, suspended position on vegetation, which enhances exposure to suitable microclimates and increases encounter rates with susceptible new hosts [1,4,5]. Such behavioral manipulation is widespread among hypocrealean entomopathogens, which infect a diverse range of insect hosts, including ants, wasps, beetles, locusts, flies, cicadas, and lepidopteran larvae [1,3,6,7,8,9].

In stark contrast, fungal pathogens of spiders, despite growing evidence of similar behavioral manipulation [10], remain critically understudied. The rapid overgrowth of somatic mycelia on spider cadavers frequently obliterates diagnostic morphological features and prevents accurate host identification. Consequently, host records are fragmentary and unreliable, host ranges are systematically underestimated, and fundamental knowledge of the ecological interactions between spiders and their fungal parasites remains largely absent [4,11].

The genus *Gibellula* (Cordycipitaceae, Hypocreales) is the most speciose and cosmopolitan group of obligate spider-pathogenic fungi [12,13]. Members of this genus are morphologically characterized by the production of one to several synnemata bearing *Aspergillus*-like (rarely *Penicillium*-like) conidiophores. These structures terminate in swollen vesicles that give rise to metulae, phialides, and unicellular, hyaline conidia [14,15]. Taxonomically, the genus has long been complicated by the separate placement of its sexual morphs in *Torrubiella*, creating persistent ambiguity between anamorphic and teleomorphic circumscriptions [11,16,17]. Following the implementation of the “one fungus, one name” nomenclatural principle and the widespread application of molecular phylogenetics, *Gibellula* is now universally accepted as the correct and sole generic name for this monophyletic clade [16,18].

According to Index Fungorum and MycoBank, more than 50 *Gibellula* species have been described to date. However, fewer than 30 of these have been included in multilocus phylogenetic analyses, and the vast majority of described species lack any molecular data [10,19,20,21,22,23,24,25,26]. As a result, the backbone phylogeny of the genus remains poorly resolved, intrageneric relationships are uncertain, and the true species diversity, particularly in tropical and subtropical regions, is almost certainly grossly underestimated [14,15,19,20,21,22,23,24,25,26]. Although integrative taxonomic approaches combining morphological observation and molecular sequence data have become standard practice in recent studies [20,21,22,23,24], molecular data are still available for only a subset of described species.

The Lancang–Mekong Basin represents a globally significant biodiversity hotspot, extending from the Hengduan Mountains in southwestern China to the lowland tropics of mainland Southeast Asia. Despite its recognized importance for fungal diversity, systematic surveys of spider-pathogenic fungi across this basin have been virtually nonexistent. During extensive field expeditions conducted from 2023 to 2025 across four countries (China, Thailand, Vietnam, and Laos), we recovered a rich assemblage of *Gibellula* specimens, including several previously undescribed taxa and numerous new national records. These collections provide a unique opportunity to fill critical knowledge gaps regarding the diversity and distribution of spider-associated fungi in Southeast Asia.

Against this background, the present study aims to: (1) formally describe four new species of *Gibellula* based on comprehensive morphological evidence and robust multilocus phylogenetic inference (nr*SSU*, ITS, nr*LSU*, *tef-1α*, *rpb1*, *rpb2*); (2) provide the first phylogenetically informed overview of *Gibellula* diversity in the Lancang–Mekong Basin, including five newly recorded species across China, Thailand, Vietnam, and Laos; and (3) synthesize an updated distributional summary and discuss the biogeographic implications of these findings, with an emphasis on the underappreciated diversity of spider-pathogenic fungi in Southeast Asia.

## 2. Materials and Methods

### 2.1. Specimen Collection and Fungus Isolation

Fungus-infected spider specimens were collected from Pu’er City, Yunnan Province, China; Đắk Lắk Province (Chu Yang Sin National Park), Vietnam; Chiang Mai Province (Maejo University), Thailand; and Oudomxay Province (Nam Kat Yorla Pa Resort), Laos during 2023–2025. Specimens were placed in sterile sampling bags in the field, and information on altitude, geographic coordinates, and habitat type was recorded for each collection site. Fungal isolation was performed using tissue isolation. Infected spider cadavers were first gently rinsed with sterile distilled water to remove surface debris, followed by surface sterilization with 75% ethanol and sterile distilled water. After drying on sterile filter paper, small pieces of fungal tissue were aseptically excised using a sterile scalpel and transferred onto potato dextrose agar (PDA; per liter: 200 g potato infusion, 20 g dextrose, 20 g agar) plates. The plates were incubated at 25 °C and checked periodically for colony development. Emerging colonies were subcultured onto fresh PDA plates to obtain pure isolates. Purified strains were maintained on PDA at 25 °C and preserved as slants at 4 °C for long-term storage. Voucher specimens and their corresponding living cultures were deposited in the Herbarium of Guizhou Medical University (GMB) and the Guizhou Medical University Culture Collection (GMBC).

### 2.2. Morphological Observations

Morphological features were evaluated based on asexual reproductive structures produced on the host. Observations spanned from macroscopic inspection to detailed microscopic examination using both dissecting and compound microscopes. Macromorphological traits, including the number, color, shape, and length of synnemata, together with the coloration of the mycelial layer covering the host, were recorded using a Nikon SMZ745T stereomicroscope (Tokyo, Japan). Micromorphology was examined by assessing the form and dimensions of vesicles, metulae, phialides, conidial heads, conidia, and conidiophores, as well as the arrangement of conidiophores on the synnematal surface. Observations and image acquisition were carried out using a Nikon ECLIPSE Ni (Nikon, Japan) equipped with a Canon EOS 700D. For detailed observation, phialides and conidia were mounted in lactophenol cotton blue. Measurements were obtained using Tarosoft Image Frame Work v.0.9.7. Cultures grown on potato dextrose agar (PDA) were additionally examined for diagnostic characters, particularly conidia and phialides.

### 2.3. DNA Extraction, Polymerase Chain Reaction (PCR), and Sequencing

DNA was extracted from both specimens and axenic cultures using the Genomic DNA Purification Kit (Qiagen GmbH, Hilden, Germany) in accordance with the manufacturer’s instructions. The ITS region was amplified with primer pair ITS5/ITS4 [27]. Partial nr*SSU* and nr*LSU* regions were generated using primer pairs nrSSU-CoF/nrSSU-CoR [28] and LR5/LR0R [29,30], respectively, while *tef-1α* was amplified with 983F/2218R [31]. Fragments of *rpb1* and *rpb2* were obtained using primer pairs RPB1-5′F/RPB1-5′R and RPB2-5′F/RPB2-5′R [13,32,33] (Table 1). PCR amplifications were conducted in 25 μL reaction mixtures consisting of 12.5 μL 2× Taq PCR Master Mix (Tiangen Biotech Co., Ltd., Beijing, China), 1 μL of each primer (10 μM), 1 μL DNA template, and 9.5 μL RNase-free water. PCR products were purified and subsequently sequenced by a commercial provider (Beijing Sinogenomax Co., Ltd., Beijing, China).

### 2.4. Phylogenetic Analyses

Taxon sampling for phylogenetic analyses was designed to represent the principal evolutionary lineages of *Gibellula* and closely related genera within Cordycipitaceae, with priority given to taxa for which multilocus sequence data were available. Sequence data for six loci (nr*SSU*, ITS, nr*LSU*, *tef-1α*, *rpb1*, and *rpb2*) were retrieved from GenBank, and corresponding taxonomic details and accession numbers are listed in Table 2. Sequence alignments were produced using MAFFT v.7 and MEGA v.7.0.26 [34], followed by manual refinement where required. Alignments of individual loci were then concatenated into a combined dataset in MEGA v.7.0.26. Phylogenetic relationships were reconstructed using both Maximum Likelihood (ML) and Bayesian Inference (BI) approaches. For ML analyses, the GTR + I + G model was applied. ML trees were generated in RAxML v.7.0.3 with branch support estimated from 1000 rapid bootstrap replicates [35]. An additional ML analysis was conducted using IQ-TREE v.2.1.3 [36], in which TIM3 + F + I + G4 was selected as the best-fit model based on the Bayesian Information Criterion (BIC), and node support was evaluated using ultrafast bootstrap approximation. For BI analyses, optimal substitution models were determined with jModelTest v.2.1.4 [37]. The GTR + I + G model was assigned to the nr*SSU*, ITS, nr*LSU*, and *tef-1α* partitions, whereas the GTR + I model was applied to *rpb1* and *rpb2*. Bayesian analyses were performed in MrBayes v.3.2.7a [38] for 5 million generations. *Blackwellomyces kaihuaensis* (HMAS 285455) and *Blackwellomyces lateris* (MFLU 18-0663) were selected as outgroup taxa. The resulting phylogenetic trees were visualized and edited using FigTree v.1.4.4 and iTOL v.7 [39].

## 3. Results

### 3.1. Sequencing and Phylogenetic Analyses

**Figure 1:**
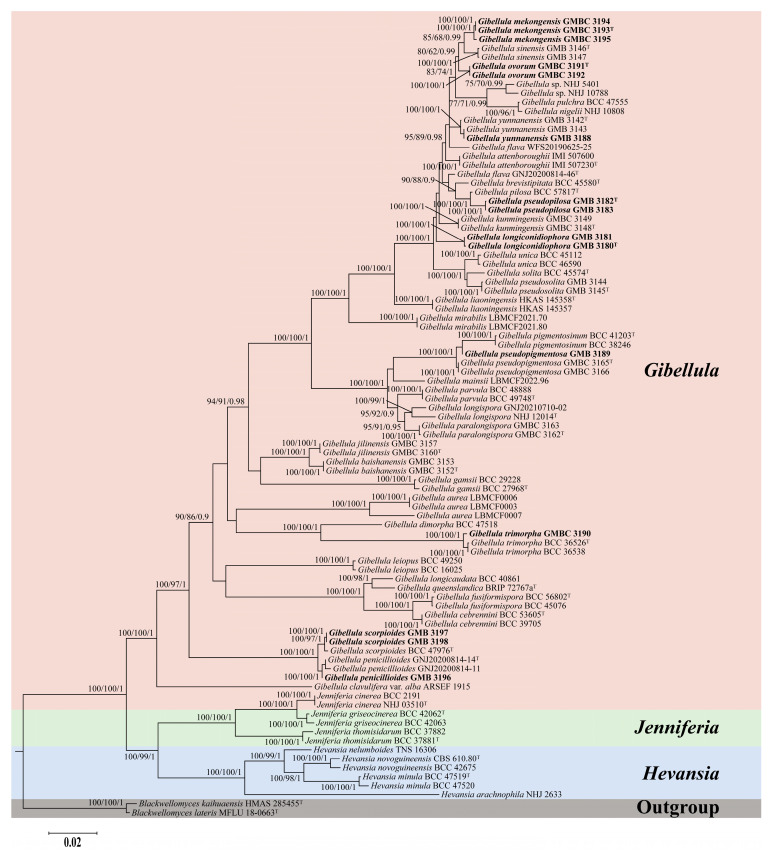
Phylogenetic tree of *Gibellula* and related genera based on a combined six-locus dataset (nr*SSU* + ITS + nr*LSU* + *tef-1α* + *rpb1* + *rpb2*). Branch support values (BS_IQ_/BS_Rax_/PP) above 75%/60%/0.9 are shown. Ex-type materials are marked with “T”. Bold labels indicate sequences generated in this study.

Phylogenetic relationships among *Gibellula* and its closely related genera were reconstructed based on a concatenated six-locus dataset (nr*SSU*, ITS, nr*LSU*, *tef-1α*, *rpb1*, and *rpb2*). The final alignment comprised 5699 base pairs, including nr*SSU* (1081 bp), ITS (780 bp), nr*LSU* (946 bp), *tef-1α* (992 bp), *rpb1* (773 bp), and *rpb2* (1127 bp). The dataset encompassed 92 fungal taxa, including 78 representatives of *Gibellula*, six of *Hevansia*, six of *Jenniferia*, and two of *Blackwellomyces*. Phylogenetic reconstructions obtained from Maximum Likelihood (ML) and Bayesian Inference (BI) analyses showed highly consistent topological structures. Node support was evaluated using ultrafast bootstrap values (BS_IQ_), RAxML bootstrap values (BS_RAx_), and Bayesian posterior probabilities (PP), and is presented throughout as BS_IQ_/BS_RAx_/PP. The analyses provided strong support for the monophyly of *Gibellula* (100%/100%/1), *Hevansia* (100%/100%/1), and *Jenniferia* (100%/100%/1). *Hevansia* and *Jenniferia* formed a strongly supported sister group (100%/99%/1), which was subsequently resolved as sister to *Gibellula* with well-support (100%/100%/1). Collectively, these three genera constituted a well-supported clade nested within Cordycipitaceae (Figure 1).

Within *Gibellula*, most taxa resolved into well-delimited, highly supported terminal clades, indicating clear species boundaries. Based on integrative morphological and phylogenetic evidence, four new species are described herein. Specimens GMB 3180 and GMB 3181 formed a strongly supported independent lineage closely clustering with *G. kunmingensis*, *G. pseudosolita*, *G. solita*, and *G. unica* (100%/100%/1). Despite its close phylogenetic affinity to these species, this lineage represents a distinct evolutionary branch with consistent morphological differences and is therefore established as *Gibellula longiconidiophora* sp. nov. Cultures of *Gibellula mekongensis* sp. nov. (GMBC 3193, GMBC 3194, GMBC 3195) formed a highly supported monophyletic lineage (100%/100%/1) and clustered with *G. sinensis* with moderate support (85%/68%/0.99). This subclade was sister to *G. ovorum* sp. nov. (GMBC 3191, GMBC 3192; 100%/100%/1) with moderate support (80%/62%/0.99), together forming a larger clade. Both novel species can be clearly distinguished from *G. sinensis* and from each other by molecular sequence divergence and morphological characteristics. Specimens GMB 3182 and GMB 3183 were resolved as a distinct lineage, *Gibellula pseudopilosa* sp. nov., which is strongly supported as the sister species to *G. pilosa* (100%/100%/1). Although phylogenetically closely related, the two species can be clearly distinguished by fixed nucleotide differences across these six nuclear loci. This molecular divergence, together with diagnostic morphological traits, robustly supports the species status of *G. pseudopilosa*.

### 3.2. Taxonomy

In this study, nine spider-associated *Gibellula* species were recognized from fungal specimens collected in the Lancang–Mekong Basin. Four taxa are described as novel species, namely *Gibellula longiconidiophora* sp. nov., *G. mekongensis* sp. nov., *G. ovorum* sp. nov., and *G. pseudopilosa* sp. nov. The other five species represent new national distributional records for the study region: *G. yunnanensis* (new record for Laos, Oudomxay Province, Nam Kat Yorla Pa Resort; voucher specimen GMB 3188, no culture obtained), *G. pseudopigmentosa* (new record for Thailand, Chiang Mai Province, Maejo University; voucher specimen GMB 3189, no culture obtained), *G. trimorpha* (new record for Vietnam, Đắk Lắk Province, Chu Yang Sin National Park; living culture GMBC 3190), *G. penicillioides* (new record for Laos, Oudomxay Province, Nam Kat Yorla Pa Resort; voucher specimen GMB 3196, no culture obtained), and *G. scorpioides* (new records for China and Laos, voucher specimens GMB 3197 from Pu’er City, Yunnan Province, China, and GMB 3198 from Oudomxay Province, Nam Kat Yorla Pa Resort, Laos; no cultures obtained). Culture accession numbers are provided for isolates with viable living cultures, while taxa without cultures were identified exclusively based on morphological examination of dried voucher specimens. Comprehensive morphological descriptions, micrographs, and taxonomic comparisons with allied species are provided for the four newly described taxa in the following sections.

*Gibellula* *longiconidiophora* Y. Wang & H. Chen, sp. nov.

Figure 2.

Mycobank No: 863473

*Etymology*. The epithet longiconidiophora refers to the long conidiophores produced by this species, from Latin longus, meaning long and conidiophorus, meaning conidium-bearing structure.

*Type*. China, Yunnan Province, Pu’er City (22.5270° N, 99.8777° E, 1125 m above sea level), on a spider from leaf litter on the forest floor, collected in August 2024 by Yao Wang (holotype: GMB 3180; ex-type living culture: not available).

*Description*. Teleomorph: Not observed. Anamorph: Mycelium yellow, covering the entire spider body and generally extending onto the legs, occasionally reaching the tarsi. Synnemata yellow, slender, cylindrical, tapering towards the apex, (2–)2.5–7(–9) mm long, formerly ca. 0.3 mm wide. Conidiophores densely aggregated, arising from the outer layer of synnemata and from hyphae attached to the host surface; short and stout, multiseptate, verrucose, (77–)182–297(–310) × (9–)10–14(–15) µm (0119 = 226 × 12 µm, *n* = 30); gradually shortening towards the synnematal apex, abruptly constricted into a distinct neck and expanding into a vesicle. Vesicles globose to subglobose, (13.5–)15–19(–20) µm ($\bar{x}$ = 18 µm, *n* = 30) in diameter, bearing a whorl of metulae. Metulae broadly obovoid, (5.5–)6.5–8(–9) × (2–)3–5(–7) µm ($\bar{x}$ = 7 × 4 μm, *n* = 30). Phialides narrowly clavate to cylindrical, (3.5–)5–7(–7.5) × 2.5–3.5 µm ($\bar{x}$ = 6 × 3 μm, *n* = 30), each producing a single conidium at the apex. Conidial heads subglobose, composed of vesicles, metulae, and phialides, (15–)17.5–38(–42.5) µm ($\bar{x}$ = 29 µm, *n* = 30) in diameter. Conidia ellipsoid to ovoid, occasionally subglobose, (1.5–)2.5–3.8(–4) × 1.2–1.8 µm ($\bar{x}$ = 3.2 × 1.5 μm, *n* = 50).

*Distribution*. Yunnan Provinces, China

*Other Material Examined*. China, Yunnan Province, Pu’er City (22.5380° N, 100.0047° E, 1615 m above sea level), on a spider from leaf litter on the forest floor, collected in August 2024 by Yao Wang (GMB 3181; living culture: not available).

*Notes*. Phylogenetically, Gibellula longiconidiophora forms a strongly supported independent lineage closely clustering with *G. kunmingensis*, *G. pseudosolita*, *G. solita*, and *G. unica* (100%/100%/1; Figure 1). This subclade is nested within a larger well-supported clade that also includes *G. attenboroughii*, *G. brevistipitata*, *G. flava*, *G. nigelii*, *G. pilosa*, *G. pseudopilosa*, *G. pulchra*, *G. sinensis*, *G. yunnanensis*, and other related species. Morphologically, *G. longiconidiophora* is a spider-parasitizing fungus characterized by yellow mycelia that entirely cover the host surface. The conidial dimensions are similar to those of *G. kunmingensis* and *G. brevistipitata*, measuring 2.5–3.8 × 1.2–1.8 μm in *G. longiconidiophora*, compared with 2–4 × 1.2–2 μm in *G. kunmingensis* and *G. brevistipitata* [44,45]. However, *G. longiconidiophora* can be clearly distinguished from other species of Gibellula by its unusually long conidiophores, measuring 182–297 × 10–14 μm, which are considerably longer than those of *G. kunmingensis* (52–120 × 14–16 μm) and *G. brevistipitata* (58–100 × 6–8 μm). The combination of its distinct phylogenetic position and morphological characteristics strongly supports the recognition of *G. longiconidiophora* as a distinct species.

*Gibellula mekongensis* Y. Wang & H. Chen, sp. nov.

Figure 3.

Mycobank No: 863474

*Etymology*. The epithet *mekongensis* refers to the Mekong River basin, where the specimens were collected.

*Type*. China, Yunnan Province, Pu’er City (23.7834° N, 100.3243° E, 1223 m above sea level), on a spider attached to the underside of a leaf, collected in August 2023 by Yao Wang (holotype: GMB 3193; ex-type living culture: GMBC 3193).

*Description*. Conidiophores sparsely arising from the mycelia covering the host, smooth to finely verrucose, (76–)82–102(–112) × (9–)10–16(–18) μm ($\bar{x}$ = 94 × 14 μm, *n* = 20). Each conidiophore terminates in a swollen vesicle bearing metulae and phialides, forming globose conidial heads (20–)35–46(–49) μm ($\bar{x}$ = 41 µm, *n* = 20) in diameter. Vesicles mostly subglobose. Metulae broadly obovoid, (6–)7–10(–12) × (3–)4–7(–9) μm ($\bar{x}$ = 8 × 5.5 μm, *n* = 20). Phialides obovoid to clavate with short necks, (8–)9–13(–15) × 3–6(–7) μm ($\bar{x}$ = 12 × 5 μm, *n* = 20). Conidia obovoid, tapering at the apex, (1.8–)2.1–2.5(–2.8) × 1–1.5(–1.8) μm ($\bar{x}$ = 2.3 × 1.2 μm, *n* = 30). *Granulomanus*-type synanamorph not observed.

*Culture characteristics*. Colonies on PDA grow slowly at 25 °C, reaching 10–12 mm in diameter after 30 days. Mycelium initially white, becoming pale yellow to grayish brown with age. Sporulation not observed in culture.

*Distribution*. Currently known from Pu’er City, Yunnan Province, China; Đắk Lắk Province, (Chu Yang Sin National Park), Vietnam; and Chiang Mai Province (Maejo University), Thailand.

*Other Material Examined*. Vietnam, Đắk Lắk Province, Chu Yang Sin National Park (12.4180° N, 108.3530° E; 900 m above sea level), on a spider attached to the underside of a leaf, collected in Jul 2023 by Yao Wang (GMB 3194; living culture: GMBC 3194); Thailand, Chiang Mai Province, Maejo University (18.8958° N, 99.0133° E; 330 m above sea level), on spiders attached to the underside of leaves, collected in August 2023 by Yao Wang (GMB 3195; living culture: GMBC 3195).

*Notes. Gibellula mekongensis* is phylogenetically placed in a moderately supported clade with *G. sinensis* (85%/68%/0.99) and forms a highly supported monophyletic lineage (100%/100%/1) together with three isolates (GMBC 3193–3195). It is sister to *G. ovorum* sp. nov. (described below) with moderate support (80%/62%/0.99). Morphologically, *G. mekongensis* resembles *G. sinensis* in conidiophore structure and globose conidial heads, but can be clearly distinguished by its shorter and broader conidiophores (82–102 × 10–16 μm vs. 92–161 × 7.7–8.5 μm) [54] and significantly smaller conidia (2.1–2.5 × 1–1.5 μm vs. 3–4.5 × 1.7–2.3 μm). The bright yellow mycelial mat covering the host in *G. mekongensis* also differs from the whitish to pale yellow mycelium reported in *G. sinensis* [54]. These stable morphological differences, in combination with multilocus phylogenetic evidence, support the recognition of *G. mekongensis* as a distinct species.

*Gibellula ovorum* Y. Wang & H. Chen, sp. nov.

Figure 4.

Mycobank No: 863475

*Etymology*. The epithet ovorum (from Latin ovum, egg, genitive plural ovorum, meaning “of eggs”) refers to the spider egg sacs from which the fungus was collected.

*Type*. Laos, Oudomxay Province, Nam Kat Yorla Pa Resort (20.8112° N, 102.8545° E, 762 m above sea level), on spider egg sacs attached to the underside of a leaf, collected in August 2023 by Yao Wang (holotype: GMB 3191; ex-type living culture: GMBC 3191).

*Description*. Teleomorph: Not observed. Anamorph: Conidiophores sparsely arising from the mycelia covering the host, smooth to finely verrucose, (112–)132.5–158.9(–165) × (9–)10–12(–12.5) μm ($\bar{x}$ = 145.4 × 11.5 μm, *n* = 20). Each conidiophore terminates in a swollen vesicle bearing metulae and phialides, forming globose conidial heads (25–)28–47(–53) μm ($\bar{x}$ = 37.5 μm, *n* = 20) in diameter. Vesicles mostly subglobose. Metulae broadly obovoid, (8.5–)10–18(–24) × (3–)5–8(–9) μm ($\bar{x}$ = 15 × 6.5 μm, *n* = 20). Phialides obovoid to clavate with short necks, (7–)9–14(–18) × 2–4 μm ($\bar{x}$ = 12.4 × 2.5 μm, *n* = 20). Conidia obovoid, tapering at the apex, (3–)3.5–4.5(–5) × 1.7–2(–2.4) μm ($\bar{x}$ = 4 × 1.9 μm, *n* = 30). *Granulomanus*-type synanamorph not observed.

*Culture characteristics*. Colonies on PDA grow slowly at 25 °C, reaching 13–15 mm in diameter after 90 days. Mycelium initially white, becoming pale yellow to grayish brown with age. Sporulation not observed in culture.

*Distribution*. Currently known only from Oudomxay Province, Laos.

*Other Material Examined*. Laos, Oudomxay Province, Nam Kat Yorla Pa Resort (20.8767° N, 102.8632° E, 744 m above sea level), on spider egg sacs attached to the underside of a leaf, collected in August 2023 by Yao Wang (GMB 3192; living culture: GMBC 3192).

*Notes*. Gibellula ovorum is phylogenetically sister to the clade comprising *G. mekongensis* and *G. sinensis* with moderate support (80%/62%/0.99; Figure 1). Morphologically, *G. ovorum* can be clearly distinguished from *G. mekongensis* by its larger conidia (3.5–4.5 × 1.7–2 μm vs. 2.1–2.5 × 1–1.5 μm) and by its unique substrate (spider egg sacs vs. adult spiders). From *G. sinensis*, *G. ovorum* differs in having broader conidiophores (10–12 μm vs. 7.7–8.5 μm) [54] and in producing a yellowish mycelial mat that covers the host, in contrast to the whitish to pale yellow mycelium of *G. sinensis* that bears abundant conidiophores [54]. These morphological differences, together with phylogenetic evidence, support the recognition of *G. ovorum* as a distinct species.

*Gibellula pseudopilosa* Y. Wang & H. Chen, sp. nov.

Figure 5.

Mycobank No: 863476

*Etymology*. The epithet pseudopilosa refers to its morphological resemblance to Gibellula pilosa, with the prefix pseudo-, meaning false.

*Type*. China, Yunnan Province, Pu’er City (22.5366° N, 100.0353° E, 1322 m above sea level), on a spider from leaf litter on the forest floor, collected in August 2024 by Yao Wang (holotype: GMB 3182; ex-type living culture: not available).

*Description*. Teleomorph: Not observed. Anamorph: Mycelium whitish-yellow, densely covering the entire spider body and usually extending onto the legs, forming a fluffy, raised mycelial layer. Synnemata yellow, slender, cylindrical, tapering towards the apex. Conidiophores densely aggregated, arising from the outer layer of synnemata and from hyphae attached to the host surface; short and stout, multiseptate, verrucose, (75–)95–131(–194) × (8–)9–12(–14) µm ($\bar{x}$ = 112 × 10 μm, *n* = 30); gradually shortening towards the synnematal apex, abruptly constricted into a distinct neck and expanding into a vesicle. Vesicles globose to subglobose, (15–)16–19(–20) µm ($\bar{x}$ = 17 µm, *n* = 30) in diameter, bearing a whorl of metulae. Metulae broadly obovoid, (6–)7–9(–11) × (1–)1.3–2 µm ($\bar{x}$ = 8 × 1.8 μm, *n* = 30). Phialides narrowly clavate to cylindrical, (4–)6–8(–9) × 2–3.4 µm ($\bar{x}$ = 6.5 × 2.5 μm, *n* = 30), each producing a single conidium at the apex. Conidial heads subglobose, composed of vesicles, metulae, and phialides, (28–)30.5–42(–50) µm ($\bar{x}$ = 38 µm, *n* = 30) in diameter. Conidia ellipsoid to ovoid, occasionally subglobose, (1.5–)2–3(–3.5) × 1.4–1.7 µm ($\bar{x}$ = 2.4 × 1.5 μm, *n* = 50).

*Distribution*. Yunnan Provinces, China.

*Other Material Examined*. China, Yunnan Province, Pu’er City (22.3865° N, 99.9385° E, 1478 m above sea level), on a spider from leaf litter on the forest floor, collected in August 2024 by Yao Wang (GMB 3183; living culture: not available).

*Notes. Gibellula pseudopilosa* is phylogenetically strongly supported as the sister species to *G. pilosa* (100%/100%/1; Figure 1). Morphologically, both species are spider-parasitizing fungi that produce mycelia completely covering the host surface [45]. However, *G. pseudopilosa* can be distinguished by its whitish-yellow mycelium, which forms a dense, fluffy, raised layer over the host, whereas *G. pilosa* has a less dense mycelial covering. Additionally, *G. pseudopilosa* typically produces multiple synnemata on a single host, while *G. pilosa* usually bears only two to three synnemata [45]. The two species also differ in conidial size: conidia of *G. pseudopilosa* measure 2–3 × 1.4–1.7 μm, notably smaller than those of *G. pilosa* (3–4 × 1.5–2 μm) [45]. These consistent morphological differences, together with phylogenetic evidence, strongly support the recognition of *G. pseudopilosa* as a distinct species.

## 4. Discussion

### 4.1. Summary of the Main Findings

In this study, we describe four new species of *Gibellula*—*G. longiconidiophora*, *G. mekongensis*, *G. ovorum*, and *G. pseudopilosa*—from the Lancang–Mekong region, together with five newly recorded species (*G. yunnanensis*, *G. pseudopigmentosa*, *G. trimorpha*, *G. penicillioides*, and *G. scorpioides*). These findings substantially expand the known diversity and geographic distribution of *Gibellula* within Cordycipitaceae. Notably, one of the new species, *G. ovorum*, represents a rare case of a *Gibellula* fungus parasitizing spider egg sacs rather than adult spiders, highlighting the previously overlooked ecological plasticity of the genus. The co-occurrence of multiple new taxa and new national records in a single study underscores both the high diversity of spider-pathogenic fungi in this biodiversity hotspot and the persistent sampling bias across many regions of Southeast and East Asia.

### 4.2. Taxonomic and Phylogenetic Significance

The integration of multilocus phylogenetic analyses (six loci: nr*SSU*, ITS, nr*LSU*, *tef-1α*, *rpb1*, *rpb2*) with detailed morphological comparisons provides robust evidence for species delimitation in *Gibellula*. The newly described species are distributed across several well-supported clades, reflecting both phylogenetic clustering and clear species-level divergence.

*Gibellula longiconidiophora* is nested within a large clade containing *G. pseudosolita*, *G. solita*, *G. unica*, *G. kunmingensis*, *G. brevistipitata*, *G. pilosa*, and related taxa, but forms a fully supported distinct lineage. Morphologically, it is readily distinguished by its exceptionally elongated conidiophores, a trait that appears evolutionarily labile and taxonomically informative. Similarly, *G. pseudopilosa* forms a strongly supported sister relationship with *G. pilosa* and can be consistently distinguished by a combination of subtle but stable features: dense whitish-yellow mycelium forming a raised hyphal layer, production of multiple synnemata on a single host, and smaller conidia. These cases illustrate that closely related species within *Gibellula* may undergo fine-scale morphological divergence, emphasizing the need for integrative taxonomy. In contrast, *G. mekongensis* and *G. ovorum* form a clade together with *G. sinensis*. They share similar conidiophore structures and globose conidial heads, reflecting close evolutionary relationships. However, they are reliably distinguished by differences in conidiophore dimensions, conidial size, and sporulation patterns on the host surface. Both *G. mekongensis* and *G. ovorum* produce a yellow mycelial mat that predominantly covers the host, with sparsely arranged conidiophores—a departure from the more typical, conidiophore-rich sporulation of *G. sinensis*. Such divergence in reproductive structures may reflect ecological or developmental differentiation and provides useful diagnostic characters. Moreover, *G. ovorum* is unique among the new species in its exclusive occurrence on spider egg sacs, a substrate preference that has rarely been documented in *Gibellula*. Overall, the strong concordance between multilocus phylogenetic evidence and stable morphological differentiation supports the recognition of the four new species. This study further demonstrates that the diversity of *Gibellula*, particularly in the Lancang–Mekong region, remains substantially underestimated, and that integrative taxonomy is essential for resolving species boundaries in this genus.

### 4.3. Biogeography and Distribution Patterns

The five newly recorded species reveal notable patterns of geographic expansion and distributional discontinuity within *Gibellula*. *Gibellula yunnanensis*, previously known only from Yunnan, China [44], is here recorded from Laos; *G. pseudopigmentosa*, originally described from Laos [54], is now documented in Thailand; *G. trimorpha*, previously known from Thailand [45], is newly recorded from Vietnam; *G. penicillioides*, previously known from Anhui Province, China [50], is newly recorded from Laos; and *G. scorpioides*, originally reported from Thailand [25], is confirmed from both China (Pu’er) and Laos. These cross-regional occurrences demonstrate that many *Gibellula* species have wider geographic distributions than previously recognized, often spanning multiple countries in Southeast and East Asia.

Such patterns suggest that the apparent geographic restriction reported in earlier studies is likely influenced by limited sampling rather than true distribution boundaries. Two main factors may account for these observations. First, *Gibellula* species may possess effective dispersal capabilities, including aerial conidial dissemination and potential host-mediated transport, facilitating long-distance movement. Second, the increasing number of new records across different countries highlights a strong sampling bias, particularly in tropical and subtropical regions where spider-associated fungi remain underexplored. Overall, these findings indicate that the Lancang–Mekong region represents a continuous biogeographic unit for spider-pathogenic fungi, rather than discrete, isolated distribution zones.

### 4.4. Ecological Implications

As obligate pathogens of spiders, *Gibellula* species may indirectly influence terrestrial food webs by affecting spider abundance and trophic interactions [59,60,61]. Field observations have reported relatively high infection rates of spiders by hypocrealean fungi in humid environments such as forests and agricultural systems [6,12,61]. Under favorable conditions, these fungi could contribute to regulating spider populations, although quantitative data on infection rates, host specificity, and ecological impact remain limited.

Among our findings, *G. ovorum* stands out as a rare example of a *Gibellula* species parasitizing spider egg sacs rather than adult spiders. This substrate shift may have profound ecological implications. Spider egg sacs represent a nutrient-rich, defenseless microhabitat that is often aggregated and spatially predictable, potentially offering a competitive advantage to fungi that can exploit this niche. To our knowledge, only a handful of spider-egg-associated fungi have been reported in Hypocreales, and *G. ovorum* is the first formally described *Gibellula* from this substrate in the Lancang–Mekong region. This discovery suggests that the ecological diversity of *Gibellula* may be broader than previously recognized, and that targeted sampling of spider egg sacs could reveal additional hidden diversity.

### 4.5. Host Association and Evolutionary Implications

Spider-pathogenic fungi in the genus *Gibellula* exhibit a high degree of host association, which likely plays a central role in their diversification. Previous studies have suggested that host specificity is a key driver of speciation in entomopathogenic fungi, particularly within Cordycipitaceae [13,62]. Although precise host identification remains challenging for many specimens, field observations indicate that *Gibellula* species often infect specific spider taxa occupying distinct ecological niches. Such host-associated differentiation may promote reproductive isolation and lead to lineage diversification. The discovery of *G. ovorum* on spider egg sacs adds a novel dimension to this host-associated diversification. Shifts to alternate substrates, such as from adult spiders to egg sacs, may represent a key innovation that reduces competition and facilitates niche partitioning. Comparative genomic or transcriptomic studies between *G. ovorum* and its close relatives (e.g., *G. mekongensis*) could uncover genetic adaptations underlying this substrate transition. The expanded distribution patterns revealed in this study provide insight into the relative roles of host distribution and fungal dispersal in shaping *Gibellula* biogeography. The occurrence of identical or closely related species across multiple countries in the Lancang–Mekong region suggests that dispersal limitation may be less restrictive than previously assumed. Instead, the continuity of suitable habitats and the widespread distribution of spider hosts may facilitate gene flow or repeated colonization events, as observed in other hypocrealean fungi [44,61].

Finally, the integration of morphological and multilocus phylogenetic data highlights the potential presence of cryptic diversity within *Gibellula*. Morphologically similar taxa may represent genetically distinct lineages, indicating that species richness in this genus is likely underestimated—a pattern consistent with broader trends in fungal systematics [17,63]. Taken together, these findings suggest that the evolution of *Gibellula* is shaped by a combination of host association, ecological factors, and historical biogeographic processes.

## Figures and Tables

**Figure 2:**
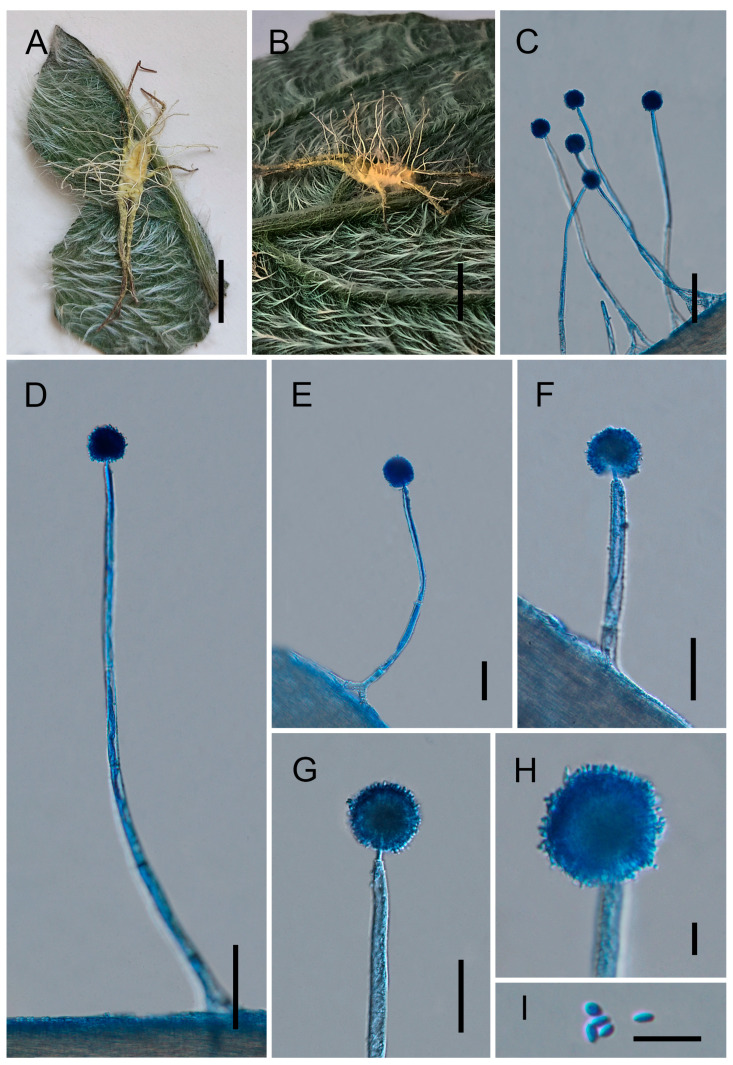
Morphology of *Gibellula longiconidiophora*. (**A**,**B**) Fungus on spider hosts. (**C**–**H**) Conidiophores showing conidial heads. (**I**) Conidia. Scale bars: 5 mm (**A**,**B**); 50 μm (**C**); 40 μm (**D**–**G**); 10 μm (**H**,**I**).

**Figure 3:**
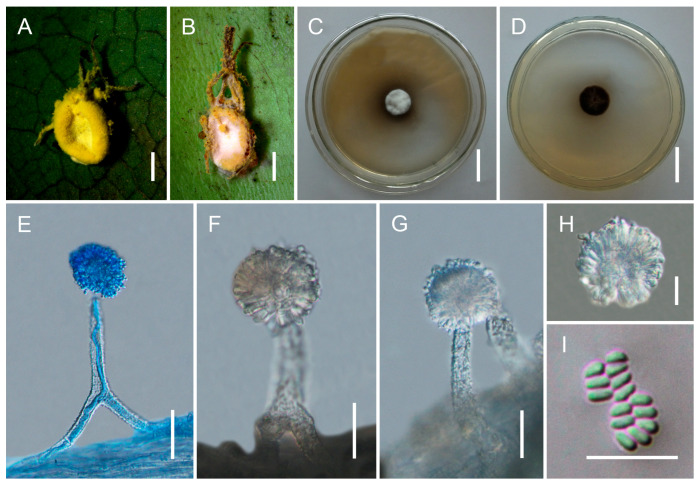
Morphology of *Gibellula mekongensis.* (**A**,**B**) Fungus on spider hosts. (**C**,**D**) Colonies on PDA (front and reverse). (**E**–**G**) Conidiophores showing conidial heads. (**H**) Conidial head. (**I**) Conidia. Scale bars: 10 mm (**A**,**B**); 20 mm (**C**,**D**); 30 µm (**E**–**G**); 10 µm (**H**,**I**).

**Figure 4:**
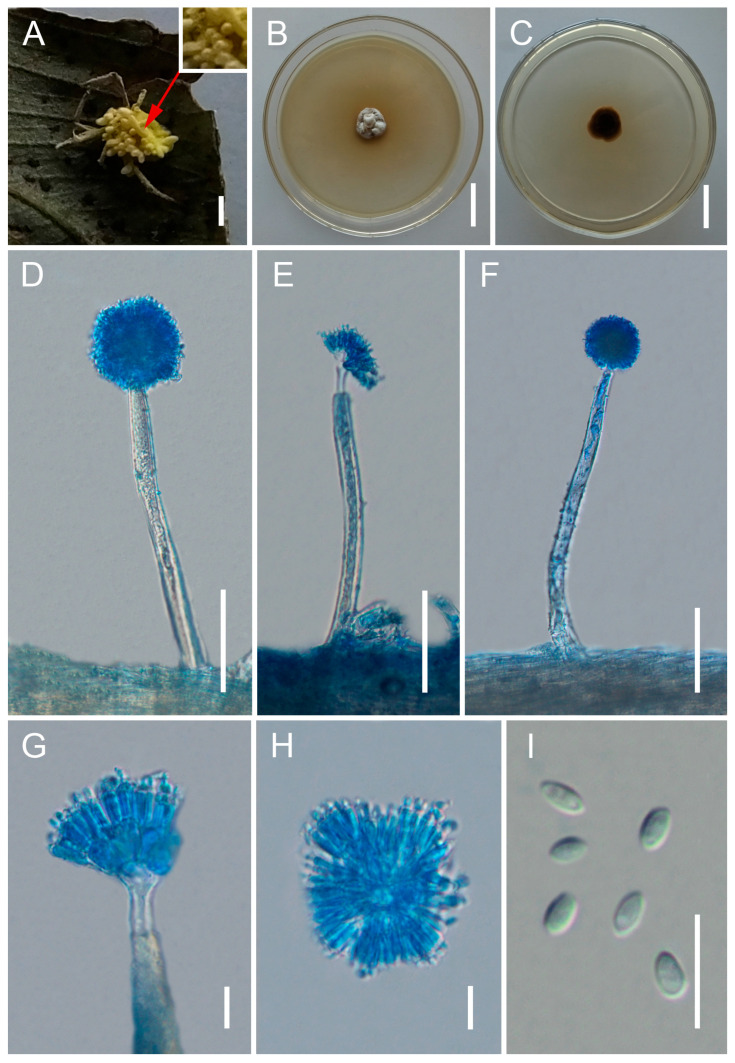
Morphology of *Gibellula ovorum.* (**A**) Fungus on spider egg sacs, covered by a dense yellowish mycelial mat. The host structure is largely obscured due to rapid fungal overgrowth. The arrow indicates the position of the egg (egg sac) beneath the mycelium. Enlarged view of the indicated region showing the emerging egg structure. (**B**,**C**) Colonies on PDA (front and reverse). (**D**–**G**) Conidiophores showing conidial heads. (**H**) Conidial head. (**I**) Conidia. Scale bars: 10 mm (**A**); 20 mm (**B**,**C**); 50 µm (**D**–**F**); 10 µm (**G**–**I**).

**Figure 5:**
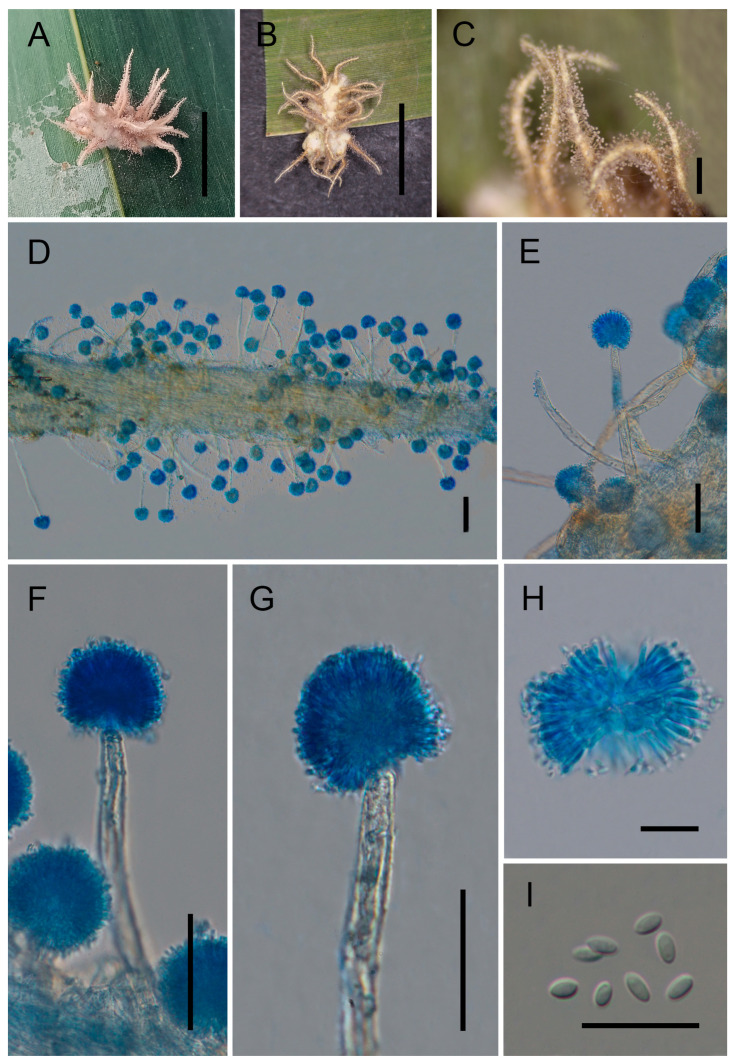
Morphology of *Gibellula pseudopilosa*. (**A**,**B**) Fungus on spider hosts. (**C**,**D**) Detail of synnemata showing conidiophores. (**E**–**G**) Conidiophores showing conidial heads. (**H**) Conidial head. (**I**) Conidia. Scale bars: 10 mm (**A**,**B**); 500 μm (**C**); 70 μm (**D**); 40 μm (**E**–**G**); 10 μm (**H**,**I**).

**Table 1:** The primer information of each gene fragment used for DNA amplification in this study.

Gene	Primer Name	Primer Sequence (5′-3′)	Reference
ITS	ITS4	TCCTCCGCTTATTGATATGC	[27]
	ITS5	GGAAGTAAAAGTCGTAACAAGG	
nr*SSU*	nrSSU-CoF	GTAGTCATATGCTTGTCTC	[28]
	nrSSU-CoR	CTTCCGTCAATTCCTTTAAG	
nr*LSU*	LR5	ACCCGCTGAACTTAAGC	[29,30]
	LR0R	ATCCTGAGGGAAACTTCG	
*tef-1α*	983F	GCTCCYGGHCAYCGTGAYTTYAT	[31]
	2218R	ATGACACCRACRGCRACRGTYTG	
*rpb1*	RPB1-5′F	CAYCCWGGYTTYATCAAGAA	[13,32,33]
	RPB1-5′R	CCNGCDATNTCRTTRTCCATRTA	
*rpb2*	fFPB2-5F	GAYGAYMGWGATCAYTTYGG	[33]
	fRPB2-7cR	CCCATRGCTTGYTTRCCCAT	

**Table 2:** Relevant species information and GenBank accession numbers for phylogenetic research in this study.

Species	Voucher/Information	GenBank Accession Number						References
		nr*SSU*	ITS	nr*LSU*	*tef-1α*	*rpb1*	*rpb2*	
*Blackwellomyces kaihuaensis*	HMAS 285455^T^	OQ981975	OQ981961	OQ981968	OQ980401	OQ980409	OQ980408	[40]
*Blackwellomyces lateris*	MFLU 18-0663^T^	MK086057	MK086059	MK086061	MK069471	MK084615	MK079354	[41]
*Gibellula agrofloretalis*	A30	PP958494	N/A	N/A	PP965288	N/A	N/A	[42]
*Gibellula agrofloretalis*	C11	PP958496	N/A	N/A	PP965293	N/A	N/A	[42]
*Gibellula agrofloretalis*	D7	PP958504	N/A	PP958435	PP965304	N/A	N/A	[40]
*Gibellula attenboroughii*	IMI 507230^T^	PQ036924	N/A	PQ036929	PQ046101	N/A	N/A	[43]
*Gibellula attenboroughii*	IMI 507600	PQ036925	PQ036927	N/A	PQ046102	N/A	N/A	[43]
*Gibellula aurea*	LBMCF0003	OK329880	N/A	N/A	OK392618	N/A	OL117022	[10]
*Gibellula aurea*	LBMCF0006	N/A	N/A	OK329875	OK392624	N/A	OK315662	[10]
*Gibellula aurea*	LBMCF0007	N/A	OK329885	OK329876	OK392622	N/A	OK315663	[10]
*Gibellula baishanensis*	GMBC 3152^T^	PX425053	PX425062	PX425069	PX434402	PX434411	PX434420	[44]
*Gibellula baishanensis*	GMBC 3153	PX425054	PX425063	PX425070	PX434403	PX434412	PX434421	[44]
*Gibellula brevistipitata*	BCC 45580^T^	N/A	OK040729	OK040706	OK040697	OK040715	N/A	[45]
*Gibellula cebrennini*	BCC 39705	N/A	MH532874	MH394673	MH521895	MH521822	MH521859	[25]
*Gibellula cebrennini*	BCC 53605^T^	N/A	MT477069	MT477062	MT503328	MT503321	MT503336	[25]
*Gibellula clavulifera* var. *alba*	ARSEF1915	DQ522562	JN049837	DQ518777	DQ522360	DQ522408	DQ522467	[46,47]
*Gibellula dimorpha*	BCC 47518	N/A	MH532884	MH394679	MH521892	MH521819	MH521863	[48]
*Gibellula flava*	GNJ20200814-46^T^	MW969660	N/A	MW969673	MW961413	MW980146	N/A	[48]
*Gibellula flava*	WFS20190625-25	MW036749	N/A	MW084343	MW091325	MW384883	N/A	[48]
*Gibellula fusiformispora*	BCC 45076	N/A	MH532882	N/A	N/A	MH521823	MH521860	[25]
*Gibellula fusiformispora*	BCC 56802^T^	N/A	MT477070	MT477063	MT503329	MT503322	MT503337	[25]
*Gibellula gamsii*	BCC 27968^T^	N/A	MH152529	MH152539	MH152560	MH152547	N/A	[23]
*Gibellula gamsii*	BCC 29228	N/A	MH152533	MH152543	MH152564	MH152551	MH152558	[23]
*Gibellula jilinensis*	GMBC 3157	PX425051	PX425060	PX425067	PX434400	PX434409	PX434418	[44]
*Gibellula jilinensis*	GMBC 3160^T^	PX425052	PX425061	PX425068	PX434401	PX434410	PX434419	[44]
*Gibellula kunmingensis*	GMBC 3148^T^	PX425057	PX425064	PX425073	PX434406	PX434415	PX434424	[44]
*Gibellula kunmingensis*	GMBC 3149	PX425058	PX425065	PX425074	PX434407	PX434416	PX434425	[44]
*Gibellula leiopus*	BCC 16025	N/A	N/A	MF416548	MF416492	MF416649	N/A	[16]
*Gibellula leiopus*	BCC 49250	N/A	OK070780	OK070781	OK070782	OK070783	OK070784	[45]
*Gibellula liaoningensis*	HKAS 145357	PQ817100	PQ817098	PQ817102	PQ815114	PQ815116	PQ815118	[49]
*Gibellula liaoningensis*	HKAS 145358^T^	PQ817099	PQ817097	PQ817101	PQ815113	PQ815115	PQ815117	[49]
*Gibellula longicaudata*	BCC 40861	N/A	OK040730	OK040707	OK040698	OK040716	OK040724	[26]
***Gibellula longiconidiophora***	**GMB 3180^T^**	**PZ144845**	**PZ144837**	**PZ144851**	**PZ150709**	**N/A**	**N/A**	**This study**
***Gibellula longiconidiophora***	**GMB 3181**	**PZ144846**	**PZ144838**	**PZ144852**	**PZ150710**	**N/A**	**N/A**	**This study**
*Gibellula longispora*	GNJ20210710-02	OL854201	N/A	OL854212	OL981628	N/A	OL981635	[50]
*Gibellula longispora*	NHJ 12014^T^	EU369098	N/A	N/A	EU369017	EU369055	EU369075	[51]
*Gibellula mainsii*	LBMCF2022.96	OQ585789	OQ589484	N/A	OQ658392	N/A	N/A	[52]
***Gibellula mekongensis***	**GMBC 3193^T^**	**PZ2592** **37**	**PZ259227**	**PZ2592** **48**	**PZ255376**	**PZ255387**	**PZ255399**	**This study**
***Gibellula mekongensis***	**GMBC 3194**	**PZ2592** **38**	**PZ2592228**	**PZ2592** **49**	**PZ255377**	**PZ255388**	**PZ255400**	**This study**
***Gibellula mekongensis***	**GMBC 3195**	**PZ259239**	**PZ2592229**	**PZ259250**	**PZ255378**	**PZ255389**	**PZ255401**	**This study**
*Gibellula mirabilis*	LBMCF2021.70	OQ585786	OQ589481	OQ585976	OQ658389	N/A	N/A	[52]
*Gibellula mirabilis*	LBMCF2021.80	OQ585787	OQ589482	OQ585977	OQ658390	N/A	N/A	[52]
*Gibellula nigelii*	NHJ 10808	EU369099	N/A	EU369035	EU369018	EU369056	EU369076	[51]
***Gibellula ovorum***	**GMBC 3191^T^**	**PZ2592** **35**	**PZ259225**	**PZ2592** **46**	**PZ255374**	**PZ255385**	**PZ255397**	**This study**
***Gibellula ovorum***	**GMBC 3192**	**PZ2592** **36**	**PZ259226**	**PZ2592** **47**	**PZ255375**	**PZ255386**	**PZ255398**	**This study**
*Gibellula paralongispora*	GMBC 3162^T^	PX425055	N/A	PX425071	PX434404	PX434413	PX434422	[44]
*Gibellula paralongispora*	GMBC 3163	PX425056	N/A	PX425072	PX434405	PX434414	PX434423	[44]
*Gibellula parvula*	BCC 48888	N/A	OK040731	OK040708	OK040699	OK040717	OK040725	[45]
*Gibellula parvula*	BCC 49748^T^	N/A	OK040732	OK040709	OK040700	OK040718	OK040726	[45]
***Gibellula penicillioides***	**GMB 3196**	**PZ2592** **40**	**PZ259230**	**PZ2592** **51**	**PZ255379**	**PZ255390**	**PZ255402**	**This study**
*Gibellula penicillioides*	GNJ20200814-11	MW969650	MW969669	MW969661	MW961415	MZ215998	N/A	[50]
*Gibellula penicillioides*	GNJ20200814-14^T^	MW969651	MW969670	MW969662	MW961416	MZ215999	N/A	[50]
*Gibellula pigmentosinum*	BCC 38246	N/A	MH532872	MH394672	MH521893	MH521800	MH521855	[26,53]
*Gibellula pigmentosinum*	BCC 41203^T^	N/A	MT477071	MT477064	MT503330	MT503323	N/A	[26]
*Gibellula pilosa*	BCC 57817^T^	N/A	OK040733	OK040710	OK040701	OK040719	N/A	[45]
***Gibellula pseudopigmentosa***	**GMB 3189**	**PZ259234**	**PZ259223**	**PZ2592** **44**	**PZ255372**	**PZ255383**	**PZ255395**	**This study**
*Gibellula pseudopigmentosa*	GMBC 3165^T^	PX624120	N/A	PX624122	PX527354	PX527350	PX527355	[54]
*Gibellula pseudopigmentosa*	GMBC 3166	PX624121	N/A	PX624123	PX527349	PX527351	PX527352	[54]
***Gibellula pseudopilosa***	**GMB 3182^T^**	**N/A**	**PZ144839**	**PZ144853**	**PZ150711**	**PZ150717**	**PZ150723**	**This study**
***Gibellula pseudopilosa***	**GMB 3183**	**N/A**	**PZ144840**	**PZ144854**	**PZ150712**	**PZ150718**	**PZ150724**	**This study**
*Gibellula pseudosolita*	GMB 3144	PX354539	PX354533	PX354545	PX370037	PX371913	PX371923	[54]
*Gibellula pseudosolita*	GMB 3145^T^	PX354540	PX354534	PX354546	PX370038	PX371914	PX371924	[54]
*Gibellula pulchra*	BCC 47555	N/A	MH532885	N/A	MH521897	MH521804	N/A	[55]
*Gibellula queenslandica*	BRIP 72767a^T^	N/A	OR452099	OR452103	OR459912	N/A	OR459907	[55]
*Gibellula scorpioides*	BCC 47976^T^	N/A	MT477078	MT477066	MT503335	MT503325	MT503339	[25]
***Gibellula scorpioides***	**GMB 3197**	**PZ2592** **41**	**PZ259231**	**PZ2592** **52**	**PZ255380**	**PZ255391**	**PZ255403**	**This study**
***Gibellula scorpioides***	**GMB 3198**	**PZ2592** **42**	**PZ259232**	**PZ2592** **53**	**PZ255381**	**PZ255392**	**PZ255404**	**This study**
*Gibellula sinensis*	GMB 3146^T^	PX354541	PX354535	PX354547	PX370039	PX371915	N/A	[54]
*Gibellula sinensis*	GMB 3147	PX354542	PX354536	PX354548	PX370040	PX371916	N/A	[54]
*Gibellula solita*	BCC 45574^T^	N/A	OK040736	OK040712	OK040703	OK040721	N/A	[45]
*Gibellula* sp.	NHJ 10788	EU369101	N/A	EU369036	EU369019	EU369058	EU369078	[51]
*Gibellula* sp.	NHJ 5401	EU369102	N/A	N/A	N/A	EU369059	EU369079	[51]
*Gibellula trimorpha*	BCC 36526^T^	N/A	OK040737	N/A	OK040704	OK040722	OK040728	[45]
*Gibellula trimorpha*	BCC 36538	N/A	MH532867	MH394668	MH521890	MH521817	MH521861	[45]
***Gibellula trimorpha***	**GMBC 3190**	**N/A**	**PZ259224**	**PZ259245**	**PZ255373**	**PZ255384**	**PZ255396**	**This study**
*Gibellula unica*	BCC 45112	N/A	OK040738	N/A	OK040705	OK040723	N/A	[45]
*Gibellula unica*	BCC 46590	N/A	MH532883	MH394678	N/A	MH521803	MH521866	[45]
*Gibellula yunnanensis*	GMB 3142^T^	PX354537	PX354531	PX354543	PX370035	PX371911	PX371921	[44]
*Gibellula yunnanensis*	GMB 3143	PX354538	PX354532	PX354544	PX370036	PX371912	PX371922	[44]
***Gibellula yunnanensis***	**GMB 3188**	**PZ259233**	**PZ259222**	**PZ259243**	**PZ255371**	**PZ255382**	**PZ255394**	**This study**
*Hevansia arachnophila*	NHJ 2633	N/A	MH532900	GQ249978	MH521917	MH521843	MH521884	[56]
*Hevansia minula*	BCC 47519^T^	N/A	MZ684087	MZ684002	MZ707811	MZ707826	MZ707833	[57]
*Hevansia minula*	BCC 47520	N/A	MZ684088	MZ684003	MZ707812	MZ707827	MZ707834	[57]
*Hevansia nelumboides*	TNS 16306	MF416585	N/A	N/A	MF416475	N/A	MF416438	[16]
*Hevansia novoguineensis*	BCC 42675	N/A	MZ684089	MZ684004	MZ707814	N/A	MZ707835	[57]
*Hevansia novoguineensis*	CBS 610.80^T^	N/A	MH532831	MH394646	MH521885	N/A	MH521844	[58]
*Jenniferia cinerea*	BCC 2191	GQ249956	GQ250000	GQ249971	GQ250029	N/A	N/A	[23]
*Jenniferia cinerea*	NHJ 03510^T^	N/A	N/A	N/A	EU369009	EU369048	EU369070	[51]
*Jenniferia griseocinerea*	BCC 42062^T^	N/A	MZ684091	MZ684006	MZ707815	MZ707828	MZ707837	[57]
*Jenniferia griseocinerea*	BCC 42063	N/A	MZ684092	MZ684007	MZ707816	MZ707829	MZ707838	[57]
*Jenniferia thomisidarum*	BCC 37881^T^	N/A	MZ684099	MZ684010	MZ707823	MZ707830	MZ707843	[57]
*Jenniferia thomisidarum*	BCC 37882	N/A	MZ684100	MZ684011	MZ707824	MZ707831	MZ707844	[57]

**Boldface:** data generated in this study; **T**: ex-type material. Institutional acronyms: **ARSEF**: Agricultural Research Service Collection of Entomopathogenic Fungal Cultures (culture collection); **BCC**: BIOTEC Culture Collection (culture collection); **CBS**: Westerdijk Fungal Biodiversity Institute (culture collection); **GMB**: Herbarium of Guizhou Medical University (herbarium); **GMBC**: Guizhou Medical University Culture Collection (culture collection); **HKAS**: Herbarium of Cryptogams, Kunming Institute of Botany, Chinese Academy of Sciences (herbarium); **IMI**: CABI Bioscience UK Centre (includes both cultures and herbarium specimens); **MFLU**: Mae Fah Luang University (herbarium); **NHJ**: National Herbarium of Japan (herbarium); **TNS**: National Museum of Nature and Science (herbarium). N/A indicating missing data.

## Data Availability

The DNA sequence data obtained in this study have been deposited in GenBank. The accession numbers can be found in the article (Table 1).
